# Tris(2,2′-bipyridine-κ^2^ *N*,*N*′)cobalt(III) octa­cyanido­tungstate(V)

**DOI:** 10.1107/S1600536809051654

**Published:** 2009-12-04

**Authors:** Qian Jun, Chi Zhang

**Affiliations:** a Molecular Materials Research Center, School of Chemical Engineering, Nanjing University of Science and Technology, 200 Xiaolingwei Road, Nanjing 210094, People’s Republic of China

## Abstract

In the title compound, [Co(C_10_H_8_N_2_)_3_][W(CN)_8_], the Co atom (..2 site symmetry) is coordinated by six N atoms from three 2,2′-bipyridine ligands in an octa­hedral geometry; the Co—N bond distances range from 1.926 (2) to 1.939 (2) Å. The W (..2 site symmetry) metal center is coordinated by eight cyanide ligands, resulting in a dodeca­hedral conformation with W—C distances in the range 1.165 (3)–2.176 (3) Å. The cations and anions are linked into a three-demensional structure by weak C—H⋯N hydrogen bonds.

## Related literature

For compounds with similar architectures, see: Przychodzeń *et al.* (2006 ▶); Withers *et al.* (2005 ▶); Mathonière *et al.* (2005 ▶). For related structures, see: Liu *et al.* (2008 ▶); Chang *et al.* (2002 ▶).
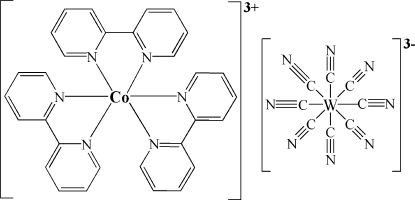


## Experimental

### 

#### Crystal data

[Co(C_10_H_8_N_2_)_3_][W(CN)_8_]
*M* *_r_* = 919.48
Orthorhombic, 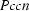

*a* = 11.465 (2) Å
*b* = 15.141 (3) Å
*c* = 20.007 (4) Å
*V* = 3473.0 (12) Å^3^
*Z* = 4
Mo *K*α radiation
μ = 3.84 mm^−1^
*T* = 250 K
0.20 × 0.20 × 0.20 mm

#### Data collection

Rigaku Mercury CCD diffractometer
Absorption correction: multi-scan (*ABSCOR*; Higashi, 1995 ▶) *T*_min_ = 0.841, *T*_max_ = 1.000
12594 measured reflections
3554 independent reflections
3023 reflections with *I* > 2σ(*I*)
*R*_int_ = 0.018

#### Refinement

*R*[*F* ^2^ > 2σ(*F* ^2^)] = 0.026
*wR*(*F* ^2^) = 0.059
*S* = 1.10
3554 reflections
245 parameters
H-atom parameters constrained
Δρ_max_ = 1.33 e Å^−3^
Δρ_min_ = −0.41 e Å^−3^

### 

Data collection: *CrystalClear* (Rigaku, 2008 ▶); cell refinement: *CrystalClear*; data reduction: *CrystalClear*; program(s) used to solve structure: *SHELXS97* (Sheldrick, 2008 ▶); program(s) used to refine structure: *SHELXL97* (Sheldrick, 2008 ▶); molecular graphics: *SHELXTL* (Sheldrick, 2008 ▶); software used to prepare material for publication: *SHELXTL*.

## Supplementary Material

Crystal structure: contains datablocks I, global. DOI: 10.1107/S1600536809051654/pv2237sup1.cif

Structure factors: contains datablocks I. DOI: 10.1107/S1600536809051654/pv2237Isup2.hkl

Additional supplementary materials:  crystallographic information; 3D view; checkCIF report

## Figures and Tables

**Table 1:** Hydrogen-bond geometry (Å, °)

*D*—H⋯*A*	*D*—H	H⋯*A*	*D*⋯*A*	*D*—H⋯*A*
C4—H4*A*⋯N4^i^	0.93	2.53	3.371 (4)	151
C10—H10*A*⋯N1^ii^	0.93	2.54	3.032 (4)	114
C12—H12*A*⋯N2^ii^	0.93	2.51	3.008 (4)	114
C1—H1*A*⋯N3	0.93	2.50	2.993 (4)	113

Symmetry codes: (i) 
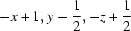
; (ii) 
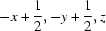
.

**Fig. 1.**
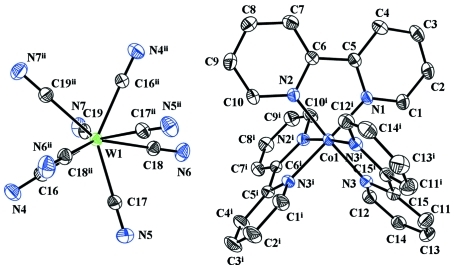
The molecular structure of a portion of the title compound, with atom labels and 30% probability displacement ellipsoids. [Symmerty codes: (i) -x + 1/2,-y + 1/2,z; (ii) -x + 3/2,-y + 1/2,z.]

**Fig. 2.**
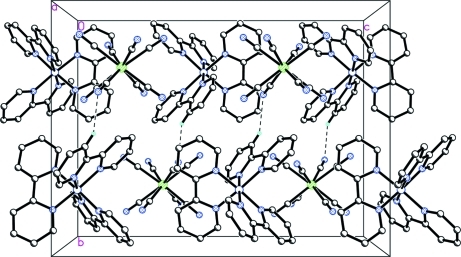
The unit cell packing diagram.

## supplementary crystallographic information

### Comment

As the direct addition of transition metal salts and [W(CN)_8_] ions (Przychodzeń *et al.*, 2006; Withers *et al.*, 2005; Mathonière *et al.*, 2005) leads to the immediate precipitation, the title compound was obtained through the interdiffusion method (Liu *et al.*, 2008; Chang *et al.*, 2002). Based on the crystal structure determination, the cations, [Co(C_10_H_8_N_2_)_3_]^2+^, and the anions, [W(CN)_8_]^3-^, have a molar ratio of 1:1. It means that the Co^2+^ of Co(ClO_4_)_2_ has been oxided into Co^3+^ during the reaction process.

As illustrated in Fig. 1, Co^3+^ has an octahedral geometry, coordinated by six nitrogen atoms from three 2,2'-bipyridine ligands. The W metal center is coordinated by eight cyanide ligands, forming a dodecahedron. The Co—N bond distances range from 1.926 (2) Å to 1.939 (2) Å, while the W—C distances in the [W(CN)_8_] unit range from 1.165 (3) to 2.176 (3) Å and C—N distances lie between 1.137 (4) Å to 1.144 (4) Å. The cations and the anions are linked with each other by weak C—H···N hydrogen bond into a three-demensional structure. (Fig. 2).

### Experimental

Co(ClO_4_)_2_ . 6H_2_O (146.4 mg, 0.4 mmol) and (Bu_3_N)_3_[W(CN)_8_] (44.72 mg, 0.1 mmol) were added into 2 ml dimethylformamide with thorough stirring for 5 minutes. After filtration, 2 ml dimethylformamide solvent and a solution of 2,2'-bipyridine (124.96 mg, 0.8 mmol) in 2 ml CH_3_OH were successively laid on the surface of the above filtrate. Red block crystals were obtained after five days.

### Refinement

H atoms were positioned geometrically and refined with riding model, with *U*_iso_ = 1.2*U*_eq_ for pyridyl H atoms, the C—H bond is 0.93 Å in 2,2'-bipyridine. The highest peak in the final difference map was located at a distance of 2.12 Å from H2A and was chemically meaningless.

### Crystal data

**Table:**

[Co(C_10_H_8_N_2_)_3_][W(CN)_8_]	*F*(000) = 1803
*M**_r_* = 919.48	*D*_x_ = 1.758 Mg m^−^^3^
Orthorhombic, *P**c**c**n*	Mo *K*α radiation, λ = 0.71073 Å
Hall symbol: -P 2ab 2ac	Cell parameters from 10230 reflections
*a* = 11.465 (2) Å	θ = 2.5–31.3°
*b* = 15.141 (3) Å	µ = 3.84 mm^−^^1^
*c* = 20.007 (4) Å	*T* = 250 K
*V* = 3473.0 (12) Å^3^	Prism, red
*Z* = 4	0.20 × 0.20 × 0.20 mm

### Data collection

**Table:**

Rigaku Mercury CCD diffractometer	3554 independent reflections
Radiation source: fine-focus sealed tube	3023 reflections with *I* > 2σ(*I*)
graphite	*R*_int_ = 0.018
Detector resolution: 28.5714 pixels mm^-1^	θ_max_ = 26.4°, θ_min_ = 2.5°
dtprofit.ref scans	*h* = −14→11
Absorption correction: multi-scan (*ABSCOR*; Higashi, 1995)	*k* = −15→18
*T*_min_ = 0.841, *T*_max_ = 1.000	*l* = −25→15
12594 measured reflections	

### Refinement

**Table:**

Refinement on *F*^2^	Primary atom site location: structure-invariant direct methods
Least-squares matrix: full	Secondary atom site location: difference Fourier map
*R*[*F*^2^ > 2σ(*F*^2^)] = 0.026	Hydrogen site location: inferred from neighbouring sites
*wR*(*F*^2^) = 0.059	H-atom parameters constrained
*S* = 1.10	*w* = 1/[σ^2^(*F*_o_^2^) + (0.0237*P*)^2^ + 2.7418*P*] where *P* = (*F*_o_^2^ + 2*F*_c_^2^)/3
3554 reflections	(Δ/σ)_max_ = 0.001
245 parameters	Δρ_max_ = 1.33 e Å^−^^3^
0 restraints	Δρ_min_ = −0.41 e Å^−^^3^

### Special details

**Table:**

Experimental. Yield: 62.1 mg in pure form, 42.1% based on Co. Analysis calculated for C_38_H_24_CoN_14_W: C 49.59, H 2.61, N 21.32%; found: C 50.31, H 2.88, N 21.44%. IR: ν, cm^-1^,2132 s.
Geometry. All e.s.d.'s (except the e.s.d. in the dihedral angle between two l.s. planes) are estimated using the full covariance matrix. The cell e.s.d.'s are taken into account individually in the estimation of e.s.d.'s in distances, angles and torsion angles; correlations between e.s.d.'s in cell parameters are only used when they are defined by crystal symmetry. An approximate (isotropic) treatment of cell e.s.d.'s is used for estimating e.s.d.'s involving l.s. planes.
Refinement. Refinement of *F*^2^ against ALL reflections. The weighted *R*-factor *wR* and goodness of fit *S* are based on *F*^2^, conventional *R*-factors *R* are based on *F*, with *F* set to zero for negative *F*^2^. The threshold expression of *F*^2^ > σ(*F*^2^) is used only for calculating *R*-factors(gt) *etc*. and is not relevant to the choice of reflections for refinement. *R*-factors based on *F*^2^ are statistically about twice as large as those based on *F*, and *R*- factors based on ALL data will be even larger.

### Fractional atomic coordinates and isotropic or equivalent isotropic displacement parameters (Å^2^)

**Table:**

	*x*	*y*	*z*	*U*_iso_*/*U*_eq_	
Co1	0.2500	0.2500	0.44279 (2)	0.02694 (12)	
N1	0.0967 (2)	0.19603 (17)	0.44129 (11)	0.0332 (5)	
N2	0.2836 (2)	0.16505 (16)	0.37377 (12)	0.0329 (5)	
N3	0.2091 (2)	0.32944 (16)	0.51483 (12)	0.0328 (5)	
C1	0.0030 (3)	0.2192 (2)	0.47655 (17)	0.0474 (8)	
H1A	0.0068	0.2693	0.5033	0.057*	
C2	−0.0986 (3)	0.1720 (3)	0.47478 (18)	0.0574 (10)	
H2A	−0.1631	0.1906	0.4992	0.069*	
C3	−0.1042 (3)	0.0979 (3)	0.43700 (18)	0.0606 (11)	
H3A	−0.1725	0.0647	0.4358	0.073*	
C4	−0.0087 (3)	0.0716 (2)	0.40038 (18)	0.0521 (9)	
H4A	−0.0114	0.0204	0.3747	0.063*	
C5	0.0911 (3)	0.1222 (2)	0.40228 (14)	0.0352 (7)	
C6	0.1962 (2)	0.10663 (18)	0.36179 (14)	0.0321 (6)	
C7	0.2071 (3)	0.0426 (2)	0.31379 (16)	0.0434 (8)	
H7A	0.1467	0.0027	0.3064	0.052*	
C8	0.3073 (3)	0.0376 (2)	0.27679 (17)	0.0479 (8)	
H8A	0.3156	−0.0056	0.2440	0.058*	
C9	0.3944 (3)	0.0961 (2)	0.28839 (17)	0.0499 (9)	
H9A	0.4628	0.0935	0.2635	0.060*	
C10	0.3813 (3)	0.1595 (2)	0.33709 (16)	0.0442 (8)	
H10A	0.4415	0.1995	0.3448	0.053*	
C12	0.1644 (3)	0.4112 (2)	0.50934 (16)	0.0399 (7)	
H12A	0.1572	0.4364	0.4671	0.048*	
C13	0.1381 (3)	0.4211 (2)	0.62628 (17)	0.0530 (9)	
H13A	0.1120	0.4514	0.6639	0.064*	
C14	0.1288 (3)	0.4588 (2)	0.56437 (16)	0.0455 (8)	
H14A	0.0989	0.5155	0.5595	0.055*	
C11	0.1858 (3)	0.3387 (2)	0.63277 (17)	0.0522 (9)	
H11A	0.1927	0.3130	0.6748	0.063*	
C15	0.2235 (3)	0.2939 (2)	0.57651 (15)	0.0369 (7)	
W1	0.7500	0.2500	0.194217 (8)	0.02995 (7)	
N4	0.9827 (3)	0.3555 (2)	0.13727 (15)	0.0565 (8)	
N5	0.8322 (3)	0.3742 (2)	0.32171 (16)	0.0571 (8)	
N6	0.5146 (3)	0.3489 (2)	0.25333 (15)	0.0590 (8)	
N7	0.6578 (3)	0.3788 (2)	0.07078 (15)	0.0569 (8)	
C16	0.9012 (3)	0.3203 (2)	0.15643 (15)	0.0398 (7)	
C17	0.8039 (3)	0.3325 (2)	0.27706 (16)	0.0401 (7)	
C18	0.5961 (3)	0.3157 (2)	0.23298 (15)	0.0397 (7)	
C19	0.6896 (3)	0.3344 (2)	0.11329 (16)	0.0396 (7)	

### Atomic displacement parameters (Å^2^)

**Table:**

	*U*^11^	*U*^22^	*U*^33^	*U*^12^	*U*^13^	*U*^23^
Co1	0.0231 (3)	0.0306 (3)	0.0271 (3)	−0.0031 (2)	0.000	0.000
N1	0.0237 (12)	0.0422 (15)	0.0337 (13)	−0.0050 (11)	0.0019 (10)	0.0019 (11)
N2	0.0287 (12)	0.0375 (14)	0.0326 (13)	−0.0011 (10)	0.0032 (10)	−0.0005 (11)
N3	0.0290 (12)	0.0350 (13)	0.0343 (13)	−0.0030 (10)	0.0011 (10)	−0.0033 (11)
C1	0.0336 (16)	0.063 (2)	0.0456 (18)	−0.0053 (16)	0.0092 (15)	−0.0064 (17)
C2	0.0346 (18)	0.081 (3)	0.057 (2)	−0.0131 (18)	0.0170 (16)	−0.009 (2)
C3	0.0351 (19)	0.080 (3)	0.067 (2)	−0.0240 (19)	0.0093 (17)	−0.004 (2)
C4	0.0433 (19)	0.055 (2)	0.058 (2)	−0.0181 (17)	0.0029 (16)	−0.0068 (18)
C5	0.0304 (15)	0.0398 (17)	0.0356 (15)	−0.0044 (13)	−0.0006 (12)	0.0055 (14)
C6	0.0301 (15)	0.0328 (15)	0.0334 (15)	−0.0011 (12)	−0.0028 (12)	0.0044 (13)
C7	0.0379 (17)	0.0415 (19)	0.051 (2)	−0.0034 (15)	−0.0036 (14)	−0.0026 (15)
C8	0.052 (2)	0.0439 (19)	0.0476 (19)	0.0035 (16)	0.0046 (17)	−0.0108 (16)
C9	0.0405 (19)	0.061 (2)	0.0481 (19)	0.0016 (17)	0.0129 (16)	−0.0079 (17)
C10	0.0307 (16)	0.053 (2)	0.0486 (19)	−0.0076 (14)	0.0094 (14)	−0.0056 (16)
C12	0.0357 (16)	0.0383 (17)	0.0457 (18)	−0.0006 (14)	0.0011 (14)	0.0009 (15)
C13	0.065 (2)	0.048 (2)	0.047 (2)	−0.0058 (18)	0.0108 (17)	−0.0167 (17)
C14	0.0397 (18)	0.0386 (18)	0.058 (2)	−0.0019 (15)	0.0057 (15)	−0.0076 (16)
C11	0.070 (3)	0.050 (2)	0.0364 (18)	−0.0059 (19)	0.0034 (17)	−0.0029 (16)
C15	0.0372 (17)	0.0398 (17)	0.0336 (15)	−0.0084 (13)	0.0014 (13)	−0.0033 (14)
W1	0.02626 (9)	0.03213 (11)	0.03147 (10)	0.00157 (7)	0.000	0.000
N4	0.0450 (17)	0.065 (2)	0.0592 (19)	−0.0143 (15)	0.0015 (15)	0.0043 (16)
N5	0.0503 (19)	0.062 (2)	0.0592 (19)	−0.0056 (16)	−0.0012 (15)	−0.0185 (16)
N6	0.0415 (17)	0.082 (2)	0.0530 (18)	0.0177 (16)	0.0023 (14)	−0.0080 (16)
N7	0.0489 (18)	0.069 (2)	0.0526 (18)	0.0133 (16)	0.0029 (14)	0.0204 (16)
C16	0.0356 (17)	0.0418 (17)	0.0421 (18)	−0.0027 (14)	−0.0030 (14)	0.0017 (15)
C17	0.0314 (16)	0.0430 (18)	0.0459 (18)	−0.0010 (14)	0.0006 (14)	−0.0029 (16)
C18	0.0346 (17)	0.0471 (19)	0.0373 (16)	0.0043 (14)	−0.0005 (13)	−0.0007 (15)
C19	0.0312 (16)	0.0458 (18)	0.0419 (17)	0.0057 (14)	0.0043 (14)	0.0032 (15)

### Geometric parameters (Å, °)

**Table:**

Co1—N2	1.926 (2)	C8—H8A	0.9300
Co1—N2^i^	1.926 (2)	C9—C10	1.376 (4)
Co1—N3^i^	1.935 (2)	C9—H9A	0.9300
Co1—N3	1.935 (2)	C10—H10A	0.9300
Co1—N1^i^	1.939 (2)	C12—C14	1.377 (4)
Co1—N1	1.939 (2)	C12—H12A	0.9300
N1—C1	1.332 (4)	C13—C14	1.368 (5)
N1—C5	1.364 (4)	C13—C11	1.368 (5)
N2—C10	1.342 (4)	C13—H13A	0.9300
N2—C6	1.358 (4)	C14—H14A	0.9300
N3—C12	1.345 (4)	C11—C15	1.384 (4)
N3—C15	1.357 (4)	C11—H11A	0.9300
C1—C2	1.367 (5)	C15—C15^i^	1.460 (6)
C1—H1A	0.9300	W1—C17	2.165 (3)
C2—C3	1.355 (5)	W1—C17^ii^	2.165 (3)
C2—H2A	0.9300	W1—C18^ii^	2.169 (3)
C3—C4	1.376 (5)	W1—C18	2.169 (3)
C3—H3A	0.9300	W1—C16	2.170 (3)
C4—C5	1.378 (4)	W1—C16^ii^	2.170 (3)
C4—H4A	0.9300	W1—C19^ii^	2.176 (3)
C5—C6	1.471 (4)	W1—C19	2.176 (3)
C6—C7	1.370 (4)	N4—C16	1.142 (4)
C7—C8	1.369 (5)	N5—C17	1.141 (4)
C7—H7A	0.9300	N6—C18	1.137 (4)
C8—C9	1.355 (5)	N7—C19	1.144 (4)
			
N2—Co1—N2^i^	88.39 (15)	N2—C10—C9	121.5 (3)
N2—Co1—N3^i^	94.04 (11)	N2—C10—H10A	119.3
N2^i^—Co1—N3^i^	176.06 (10)	C9—C10—H10A	119.3
N2—Co1—N3	176.06 (10)	N3—C12—C14	121.9 (3)
N2^i^—Co1—N3	94.04 (11)	N3—C12—H12A	119.0
N3^i^—Co1—N3	83.70 (15)	C14—C12—H12A	119.0
N2—Co1—N1^i^	95.11 (10)	C14—C13—C11	119.8 (3)
N2^i^—Co1—N1^i^	83.60 (10)	C14—C13—H13A	120.1
N3^i^—Co1—N1^i^	93.09 (10)	C11—C13—H13A	120.1
N3—Co1—N1^i^	88.24 (10)	C13—C14—C12	118.9 (3)
N2—Co1—N1	83.60 (10)	C13—C14—H14A	120.6
N2^i^—Co1—N1	95.11 (10)	C12—C14—H14A	120.6
N3^i^—Co1—N1	88.24 (10)	C13—C11—C15	119.7 (3)
N3—Co1—N1	93.09 (10)	C13—C11—H11A	120.2
N1^i^—Co1—N1	178.22 (13)	C15—C11—H11A	120.2
C1—N1—C5	118.7 (3)	N3—C15—C11	120.5 (3)
C1—N1—Co1	127.7 (2)	N3—C15—C15^i^	114.32 (17)
C5—N1—Co1	113.36 (19)	C11—C15—C15^i^	125.2 (2)
C10—N2—C6	118.6 (3)	C17—W1—C17^ii^	80.10 (17)
C10—N2—Co1	126.9 (2)	C17—W1—C18^ii^	76.03 (12)
C6—N2—Co1	114.46 (19)	C17^ii^—W1—C18^ii^	72.18 (12)
C12—N3—C15	119.1 (3)	C17—W1—C18	72.18 (12)
C12—N3—Co1	127.2 (2)	C17^ii^—W1—C18	76.03 (12)
C15—N3—Co1	113.7 (2)	C18^ii^—W1—C18	138.10 (16)
N1—C1—C2	122.4 (3)	C17—W1—C16	75.86 (12)
N1—C1—H1A	118.8	C17^ii^—W1—C16	141.03 (12)
C2—C1—H1A	118.8	C18^ii^—W1—C16	72.52 (12)
C3—C2—C1	119.2 (3)	C18—W1—C16	123.33 (12)
C3—C2—H2A	120.4	C17—W1—C16^ii^	141.03 (12)
C1—C2—H2A	120.4	C17^ii^—W1—C16^ii^	75.86 (12)
C2—C3—C4	119.9 (3)	C18^ii^—W1—C16^ii^	123.33 (12)
C2—C3—H3A	120.0	C18—W1—C16^ii^	72.52 (12)
C4—C3—H3A	120.0	C16—W1—C16^ii^	139.23 (16)
C3—C4—C5	119.0 (3)	C17—W1—C19^ii^	144.84 (12)
C3—C4—H4A	120.5	C17^ii^—W1—C19^ii^	108.76 (12)
C5—C4—H4A	120.5	C18^ii^—W1—C19^ii^	74.80 (11)
N1—C5—C4	120.7 (3)	C18—W1—C19^ii^	142.59 (11)
N1—C5—C6	114.1 (2)	C16—W1—C19^ii^	76.99 (12)
C4—C5—C6	125.1 (3)	C16^ii^—W1—C19^ii^	72.95 (11)
N2—C6—C7	121.1 (3)	C17—W1—C19	108.76 (12)
N2—C6—C5	113.7 (3)	C17^ii^—W1—C19	144.84 (12)
C7—C6—C5	125.1 (3)	C18^ii^—W1—C19	142.59 (11)
C8—C7—C6	119.6 (3)	C18—W1—C19	74.80 (11)
C8—C7—H7A	120.2	C16—W1—C19	72.95 (11)
C6—C7—H7A	120.2	C16^ii^—W1—C19	76.99 (12)
C9—C8—C7	119.3 (3)	C19^ii^—W1—C19	83.84 (16)
C9—C8—H8A	120.3	N4—C16—W1	178.2 (3)
C7—C8—H8A	120.3	N5—C17—W1	178.3 (3)
C8—C9—C10	119.8 (3)	N6—C18—W1	178.9 (3)
C8—C9—H9A	120.1	N7—C19—W1	179.9 (4)
C10—C9—H9A	120.1		

Symmetry codes: (i) −*x*+1/2, −*y*+1/2, *z*; (ii) −*x*+3/2, −*y*+1/2, *z*.

### Hydrogen-bond geometry (Å, °)

**Table:**

*D*—H···*A*	*D*—H	H···*A*	*D*···*A*	*D*—H···*A*
C4—H4A···N4^iii^	0.93	2.53	3.371 (4)	151
C10—H10A···N1^iv^	0.93	2.54	3.032 (4)	114
C12—H12A···N2^iv^	0.93	2.51	3.008 (4)	114
C1—H1A···N3	0.93	2.50	2.993 (4)	113

Symmetry codes: (iii) −*x*+1, *y*−1/2, −*z*+1/2; (iv) −*x*+1/2, −*y*+1/2, *z*.
